# Molecular Genetics Diversity of Primary Hemophagocytic Lymphohistiocytosis among Polish Pediatric Patients

**DOI:** 10.1007/s00005-021-00635-4

**Published:** 2021-10-22

**Authors:** Katarzyna Bąbol-Pokora, Magdalena Wołowiec, Katarzyna Popko, Aleksandra Jaworowska, Yenan T. Bryceson, Bianca Tesi, Jan-Inge Henter, Wojciech Młynarski, Wanda Badowska, Walentyna Balwierz, Katarzyna Drabko, Krzysztof Kałwak, Lucyna Maciejka-Kembłowska, Anna Pieczonka, Grażyna Sobol-Milejska, Sylwia Kołtan, Iwona Malinowska, for the Polish Pediatric Hematology, Oncology Society

**Affiliations:** 1 Department of Pediatrics, Oncology and Hematology, Medical University of Lodz, Lodz, Poland; 2 Department of Pediatrics, Hematology and Oncology, Medical University of Warsaw, Żwirki i Wigury 63A, 02-091 Warsaw, Poland; 3 Department of Laboratory Diagnostics and Clinical Immunology of Developmental Age, Medical University of Warsaw, Warsaw, Poland; 4 Department of Medicine, Centre for Hematology and Regenerative Medicine, Karolinska Institutet, Karolinska University Hospital Huddinge, Stockholm, Sweden; 5 Division of Pediatric Hematology and Oncology, Children Hospital, Olsztyn, Poland; 6 Department of Pediatrics Oncology and Hematology, University Children’s Hospital, Jagiellonian University Collegium Medicum, Krakow, Poland; 7 Department of Pediatric Hematology, Oncology and Stem Cell Transplantation, Medical University of Lublin, Lublin, Poland; 8 Department of Pediatric Stem Cell Transplantation, Hematology and Oncology, Medical University, Wroclaw, Poland; 9 Department of Pediatrics, Hematology and Oncology, Medical University of Gdansk, Gdansk, Poland; 10 Department of Pediatric Oncology, Hematology and Transplantology, University of Medical Sciences, Poznan, Poland; 11 Department of Pediatrics, Hematology and Oncology, Medical University of Silesia, Silesia, Poland; 12 Department of Pediatrics, Hematology and Oncology, Nicolaus Copernicus University, Collegium Medicum in Bydgoszcz, Bydgoszcz, Poland

**Keywords:** Hemophagocytic lymphohistiocytosis, Genetics, Novel variants, Clinical course

## Abstract

Hemophagocytic lymphohistiocytosis (HLH) is a clinical syndrome of life-threatening inflammation caused by an excessive, prolonged and ineffective immune response. An increasing number of HLH cases is recognized in Poland, but the genetic causes of familial HLH (FHL) have not been reported. We investigated the molecular genetics and associated outcomes of pediatric patients who met HLH criteria. We studied 54 patients with HLH, 36 of whom received genetic studies. Twenty-five patients were subjected to direct sequencing of the *PRF1*, *UNC13D*, *STX11, XIAP* and *SH2D1A* genes. Additionally, 11 patients were subjected to targeted next-generation sequencing. In our study group, 17 patients (31%) were diagnosed with primary HLH, with bi-allelic FHL variants identified in 13 (36%) patients whereas hemizygous changes were identified in 4 patients with X-linked lymphoproliferative diseases. In addition, one patient was diagnosed with X-linked immunodeficiency with magnesium defect, Epstein–Barr virus infection and neoplasia due to a hemizygous *MAGT1* variant; another newborn was diagnosed with auto-inflammatory syndrome caused by *MVK* variants. The majority (65%) of FHL patients carried *UNC13D* pathogenic variants, whereas *PRF1* variants occurred in two patients. Novel variants in *UNC13D*, *PRF1* and *XIAP* were detected*.* Epstein–Barr virus was the most common trigger noted in 23 (65%) of the patients with secondary HLH. In three patients with secondary HLH, heterozygous variants of FHL genes were found. Overall survival for the entire study group was 74% with a median of 3.6 years of follow-up. Our results highlight the diversity of molecular causes of primary HLH in Poland.

## Introduction

Hemophagocytic lymphohistiocytosis (HLH) is a clinical syndrome of life-threatening inflammation caused by an excessive, prolonged and ineffective activation of the immune cells caused by various underlying factors. The pathway of HLH pathogenesis involves excessive activation of CD8^+^ T lymphocytes and macrophages leading to hypercytokinemia (Henter et al. 1998). HLH is characterized by fever, severe cytopenia, coagulation defects, organomegaly (spleen and liver), hyperferritinemia, liver function impairment and frequently central nervous system (CNS) involvement (Henter et al. 2007; Janka 2012; Jordan et al. 2011; Madkaikar et al. 2016; Malinowska et al. 2014). Diagnosis of HLH typically requires the fulfillment of at least five of eight clinical and laboratory criteria (Henter et al. 2007).

Depending on the etiology, HLH can be divided into genetic (FHL: familial HLH) and acquired (sHLH: secondary HLH) forms (Henter et al. 2007; Janka 2012). According to the Histiocyte Society classification of histiocytic disorders, primary HLH is associated with several Mendelian inherited immune disorders, including defects in lymphocyte cytotoxicity (FHL, XLP1), pigmentation disorders, such as Griscelli syndrome 2, Chédiak–Higashi syndrome, and Hermansky–Pudlak syndrome type II, as well as HLH related to abnormalities of inflammasome activation: XLP2, *NLRC4*, and *CDC42* changes (Emile et al. 2016; Lam et al. 2019). FHL is caused by autosomal recessive pathogenic changes in genes that encode proteins required for NK cell and CD8^+^ T lymphocyte cytotoxicity. FHL has been divided into five subtypes, FHL1 to FHL5. FHL1 was mapped to chromosome 9q21.3–q22 (OMIM #603552) based on the linkage analysis of four consanguineous Pakistani families with FHL (Ohadi et al. 1999), but a causative gene has not been identified. FHL2 (OMIM #603553) is caused by pathogenic variants in *PRF1* encoding perforin. Perforin is the component of cytotoxic cell granules responsible for target cell entry of granzymes, which are serine proteases that promote apoptosis in target cells. FHL2 is associated with a diminished perforin expression in cytotoxic granules, leading to defective cytotoxicity (Molleran Lee 2004). FHL3 (OMIM #608898) is caused by genetic defects in *UNC13D*, encoding Munc13-4, the priming factor at the immunological synapse, required for the release of the lytic granule contents (Feldmann et al. 2003). Munc13-4 deficiency can be identified both by the cytotoxicity assay and the degranulation test (Molleran Lee 2004). FHL4 (OMIM #603552) is caused by the bi-allelic changes in STX11, encoding syntaxin-11. Syntaxin-11 is also involved in membrane fusion events and required for the release of the cytotoxic granule contents. Syntaxin-11 deficiency results in impaired cytotoxic activity of NK cells (Bryceson et al. 2007; zur Stadt et al. 2005). FHL5 is due to defects in *STXBP2* (OMIM #613101), encoding Munc 18–2, a protein that plays an important role in membrane fusion (zur Stadt et al. 2009). Munc 18–2 interacts with syntaxin-11, hence similarly, Munc 18–2 deficient cells have defective cytotoxic activity (Côte et al. 2009).

Besides FHL, where HLH is the primary disease manifestation, other forms of primary HLH may display additional manifestations (Janka 2012). HLH is associated with rare hereditary pigmentary disorders, i.e., Griscelli syndrome 2, Chédiak–Higashi syndrome, and Hermansky–Pudlak syndrome type II, caused by autosomal recessive changes in *RAB27A, LYST* and *AP3B1*, respectively (Elstak et al. 2011; Lozano et al. 2014; Sieni et al. 2014). Furthermore, X-linked lymphoproliferative diseases: XLP1 (caused by defects of *SH2D1A* (OMIM #300490), encoding SLAM-associated protein (SAP) [19]) and XLP2 (caused by changes in *XIAP*, which encodes the X-linked inhibitor of apoptosis (OMIM #300079) (Sieni et al. 2014) also frequently develop HLH. X-linked immunodeficiency with magnesium defect, Epstein–Barr virus (EBV) infection and neoplasia (XMEN) is another X-linked immunodeficiency caused by pathogenic variants in *MAGT1* (OMIM #300715), encoding a magnesium channel required for the control of EBV infection and neoplasia (Li et al. 2014). HLH has also been associated with other Mendelian disorders affecting inflammation, including lysinuric protein intolerance caused by *SLC7A7* defects, and CD27 and ITK deficiency caused by autosomal recessive changes in *CD27* and *ITK*, respectively (Ghosh et al. 2018, 2020).

HLH is a rare disease with an estimated yearly incidence (equivalent to the prevalence) of 0.1/10,000 when accounting for both primary and secondary forms (Henter et al. 1991; Meeths et al. 2011). The incidence of HLH in Swedish children is 1.2 cases per million per year, but it is believed that these figures are underestimated. An increasing number of HLH cases is recognized in Poland, nevertheless, no data on the genetic background of primary HLH have been published so far. We present the molecular genetics among Polish pediatric HLH patients. Clinical and laboratory findings, results of genetic sequencing, treatment method and outcome were derived from the Polish Registry of HLH for the Polish Society of Oncology and Hematology.

## Materials and Methods

### Subjects

Fifty-four patients hospitalized in the Department of Pediatrics, Hematology and Oncology or in 12 collaborating referral centers in Poland between September 2008 and September 2019, all meeting five or more HLH-2004 diagnostic criteria, were included in the study. Informed written consent and approval of the Ethics Committee of the Medical University of Warsaw was obtained.

Consanguinity between parents was not reported in any patient. Family history of HLH was reported in four patients.

### HLH Criteria and Additional Immunological Tests

These eight criteria are: (i) fever; (ii) splenomegaly; (iii) cytopenia of two or more cell lines (hemoglobin ≤ 90 g/L, platelets ≤ 100 × 10^9^/L, neutrophils ≤ 1 × 10^9^/L); (iv) hypofibrinogenemia (≤ 1.5 g/L) or hypertriglyceridemia (≥ 265 mg/dL); (v) hyperferritinemia (≥ 500 ng/mL); (vi) increased level of soluble CD25 (sCD25, ≥ 2400 U/mL); (vii) evidence of hemophagocytosis; and (viii) decreased or absent NK cell cytotoxicity.

Measurements of perforin expression, granule release assays, cytotoxicity assays were performed, as previously described (Bryceson et al. 2007; Marsh et al. 2010; Schneider et al. 2002).

### Molecular Tests

Molecular analyses were performed either by direct Sanger sequencing (25 patients treated in 2008–2015) or by Next-Generation Sequencing (11 patients treated in 2015–2019). The study was conducted at Karolinska Institute (Sweden), and in the Laboratory of Immunopathology and Genetics in Lodz (Poland).

### Sanger Sequencing

Peripheral blood samples were analyzed by Sanger sequencing, starting with the most frequently defective *PRF1* gene, and in cases of negative results direct sequencing of the *UNC13D* and *STX11* genes. Standard PCR conditions were used with the primers specifically designed to analyze variants using Primer 3 v. 0.4.0 (Untergasser et al. 2012). Products were sequenced on an ABI3130 4-capillary sequencer (Thermo Fisher Scientific) and the results were analyzed using Sequencher v. 5.0.

### Next-Generation Sequencing

Targeted next-generation sequencing (NGS) was performed using either the TruSight One panel (Illumina, USA) or the custom designed SureSelect QXT panel (Agilent Technologies Inc., Santa Clara, CA, USA) which included 535 genes related to hematological diseases.

The sequencing libraries were prepared after DNA extraction from peripheral blood samples using Flexi Gene DNA Kit (Qiagen, Germany). The samples were checked for quality using Qubit v.3 (Thermo Fisher Scientific). Sequencing libraries were prepared according to the manufacturer’s protocols (Agilent Technologies: https://www.agilent.com/en/product/next-generation-sequencing/amplicon-based-next-generation-sequencing-ngs; Illumina: https://www.illumina.com/products/by-type/clinical-research-products/trusight-one.html). High-throughput sequencing was performed on NextSeq550 (Illumina, USA) in the process of 300 bp paired-end run using Mid Output Kit (Illumina, USA). The data analyses of the target regions were performed using Burrows–Wheeler Aligner Genome Alignment Software and the GATK Variant Caller algorithms and mapped to the human genome reference sequence GRCh37/hg19 (Li and Durbin 2010). The results were next analyzed using Variant Studio v. 3.0 (Illumina, USA) and Integrative Genomics Viewer v.2.3 (Robinson et al. 2011). Sequence analysis initially focused on the genes related to congenital HLH (Table 1). The filtering criteria included coverage with at least 20 reads and a minor allele frequency below 0.01 in 1000 Genomes, GnomAD and ExAC databases. All filtered variants were investigated by several bioinformatics tools: SIFT, Mutation Taster, and PolyPhen-2 (Adzhubei et al. 2010; Higashi et al. 1954; Ng and Henikoff 2001; Schwarz et al. 2014). The pathogenicity of the revealed variants was estimated based on ClinVar, ExAC, OMIM, HGMD, Varsome and LOVD databases (Fokkema et al. 2011; Kopanos et al. 2018; Landrum et al. 2016; Lek et al. 2016; McKusick 2007; Stenson et al. 2014; Untergasser et al. 2012) according to ACMG classification rules (Richards et al. 2015). An internal database was also used to filter out the recurrent variants.

**Table 1:** Sequence analysis initially focused on the genes related to congenital HLH

Gene	Chr	Phenotype	Inheritance pattern	OMIM
*PRF1*	10q22.1	FHL2	AR	603,553
*UNC13D*	17q25.1	FHL3	AR	608,898
*STX11*	6q24.2	FHL4	AR	603,552
*STXBP2*	19p13.2	FHL5	AR	613,101
*RAB27A*	15q21.3	GS2	AR	603,868
*SH2D1A*	Xq25	XLP1	XL	308,240
*XIAP*	Xq25	XLP2	XL	300,635
*MAGT1*	Xq21.1	XMEN	XL	300,853
*AP3B1*	5q14.1	HPS2	AR	608,233
*ITK*	5q33.3	LPFS1	AR	186,973
*CD27*	12p13.31	LPFS2	AR	186,711
*LYST*	1q42.3	CHS	AR	606,897
*NLRC4*	2p22.3	AIFEC	AD	606,831

Patients in whom pathogenic or likely pathogenic variants were identified, as well as their family members, were additionally subjected to Sanger sequencing.

### Statistics

Statistical analysis was performed using statistics SPSS, V18 Software. Probability of survival from diagnosis to end of follow-up was estimated using the Kaplan–Meier life table method.

## Results

### Patient Characteristics

All eight HLH criteria were evaluated in 39 patients, while seven criteria were analyzed in 53 patients. Fifty-three patients presented five criteria and one patient was qualified based on presence of typical HLH mutation. No clinical and laboratory data except result of sequencing and date of death were available for this female infant with *UNC13D* mutation. Patients with macrophage activation syndrome and neoplastic diseases were excluded from the study. The clinical characteristics of patients are summarized in Table 2.

**Table 2:** Characteristics of Polish patients with HLH

	*n* = 54	sHLH *n* = 35	FHL *n* = 13	XLP1 and XLP2 *n* = 4	XMEN *n* = 1	MKD *n* = 1
Age at diagnosis (year)						
Median	2.87	5.56	0.24	3.75	17	Started as intrauterine presentation
Average	4.7	6.6	0.2	3.42		
Sd	4.83	4.82	0.5	1.62		
Range	0.01–17.85	0.47–17.85	0.01–0.82	1.25–4.93		
Sex (M/F)	30/24	18/17	7/6	4/0	1/0	0/1
Diagnostic criteria						
Fever	53/53 (100%)	35/35(100%)	12/12(100%)	4/4(100%)	No	Yes
Splenomegaly	51/53 (96%)	33/35 (94%)	12/12 (100%)	4/4 (100%)	Yes	Yes
Bicytopenia	51/53 (96%)	33/35 (94%)	12/12 (100%)	4/4 (100%)	No	Yes
Triglycerides > 265 mg/dl	38/51 (75%)	25/33 (76%)	9/12 (75%)	2/4 (50%)	No	No
Fibrinogen < 150 mg/dl	37/53 (70%)	25/35 (71%)	7/12 (58%)	3/4 (75%)	No	No
Ferritin > 500 /dl	50/53 (94%)	33/35 (94%)	12/12 (100%)	3/4 (75%)	No	Yes
NK cell activity low	28/40 (70%)	19/26 (76%)	8/11 (73%)	0/2 (100%)	Not done	Yes
sCD25 > 2400 U/l	6/6 (100%)	0/0	4/4 (100%)	1/1 (100%)	Not done	Yes
Hemophagocytosis	39/52 (75%)	29/34 (85%)	7/12 (58%)	1/4 (25%)	No	Yes
CNS involvement	17/51 (33%)	8/34 (24%)	6/12 (50%)	3/3 (100%)	No	No
Neurological symptoms	8/17	6/8	2/6	0/3		
CSF pleocytosis	13/17	5/8	5/6	3/3		
CSF proteinosis	7/17	3/8	4/6	0/3		
MRI abnormalities	7/15	4/8	2/6	1/1		

*CSF* cerebrospinal fluid, *MRI* magnetic resonance imaging

### Variation Analysis

Genetic studies were performed in 36 patients, among whom 25 patients were subjected to direct sequencing of the *PRF1*, *UNC13D* and *STX11* genes and eleven patients were subjected to targeted NGS followed by Sanger sequencing.

Out of 36 patients, pathogenic or likely pathogenic variants were identified in 19 patients, of whom 13 carried variants in FHL genes (*PRF1* and *UNC13D),* four patients carried variants in XLP genes *(XIAP* and *SH2D1A),* one had a pathogenic variant in *MAGT1* and one a homozygous nonsense variant in *MVK*. The results of genetic testing are presented in Table 3. Genetic testing was not performed in 18 patients for various reason, including lack of qualification for genetic studies at the center based on normal results of immunological tests for cytolytic function as well as lack of testing availability at the time of evaluation.

**Table 3:** Patients with pathogenic or likely pathogenic genetic variants

Patient	Sex	Age (year)	SCT	Outcome	Functional defect	pHLH	Gene	Transcript	Genotype	Inheritance	Change	DNA HGVS	Protein HGVS	ACMG classification	dbSNP	GnomAD AF	References
Patients with biallelic defects																	
1	M	0.03	No	Death	Nd	FHL3	*UNC13D*	NM_199242.2	hmz	Both	Frameshift	c.2346_2349del	p.Arg782Serfs*12	Pathogenic	rs764196809	1.03*e*−4	zur Stadt et al. (2006)
2	F	0.02	Yes	Alive	not detected	FHL3	*UNC13D*	NM_199242.2	hmz	Both	Frameshift	c.2346_2349del	p.Arg782Serfs*12	Pathogenic	rs764196809	1.03*e*−4	zur Stadt et al. (2006)
3	M	0.01	Yes	Death	low NK activity, abnormal degranulation	FHL3	*UNC13D*	NM_199242.2	hmz	Both	Splice-site	c.753 + 1G > T	p.Ile229Thrfs*35	Pathogenic	rs201908137	4.38*e*−5	Alsina et al. (2014)
4	M	0.82	Yes	Death	low NK activity	FHL3	*UNC13D*	NM_199242.2	Compound	Paternal	Nonsense	c.640C > T	p.Arg214*	Pathogenic	rs769243366	3.98*e*−6	Yamamoto et al. (2004)
					abnormal degranulation				htz	Maternal	Frameshift	c.2346_2349del	p.Arg782Serfs*12	Pathogenic	rs764196809	1.03*e*−4	zur Stadt et al. (2006)
5	M	0.39	Yes	Alive	low NK activity, abnormal degranulation	FHL3	*UNC13D*	NM_199242.2	hmz	Both	Splice-site	c.753 + 1G > T	p.Ile229Thrfs*35	Pathogenic	rs201908137	4.38*e*−5	Alsina et al. (2014)
6	M	0.31	Yes	Alive	low NK activity	FHL3	*UNC13D*	NM_199242.2	Compound	Maternal	Nonsense	c.551G > A	p.Trp184*	Pathogenic	rs754292065	1.07*e*−5	Murphy et al. (2010)
					abnormal degranulation				htz	Paternal	Missense	c.3049G > A	p.Glu1017Lys	Likely pathogenic	rs776737156	4.35*e*−6	Sieni et al. (2011)
7	F	0.22	No	Death	not detected	FHL3	*UNC13D*	NM_199242.2	compound htz	Paternal	Nonsense	c.247C > T	p.Arg83*	Pathogenic	rs1274685768	4.01*e*−6	Rudd et al. (2008)
										Maternal	Splice-site	2710-1G > A	p.?	Pathogenic	Novel	nd	nd
8	F	0.08	Yes	Alive	low NK activity, abnormal degranulation	FHL3	*UNC13D*	NM_199242.2	hmz	Both	Frameshift	c.2346_2349del	p.Arg782Serfs*12	Pathogenic	rs764196809	1.03*e*−4	zur Stadt et al. (2006)
9	M	0.16	Yes	Alive	not detected	FHL3	*UNC13D*	NM_199242.2	Compound htz	Paternal	Splice-site	c.569 + 1G > A	p.?	Pathogenic	rs1400391434	4.02*e*−6	nd
										Maternal	Frameshift	c.2346_2349del	p.Arg782Serfs*12	Pathogenic	rs764196809	1.03*e*−4	zur Stadt et al. (2006)
10	F	0.2	Yes	Death	absent perforin, low NK activity	FHL2	*PRF1*	NM_005041.2	Compound htz	Paternal	Frameshift,	c.808_812del	p.Gly270Hisfs*9	Pathogenic	Novel	nd	Maxwell et al. (2016) and Pronicka et al. (2016)
										Maternal	Missense	c.938A > T	p.Asp313Val	Likely pathogenic	rs755737064	1.19*e*−5	nd
11	F	0.2	Yes	Alive	Absent perforin, low NK activity	FHL2	*PRF1*	NM_005041.2	Compound htz	Paternal	Frameshift,	c.284G > T	p.Trp95Leu	Likely pathogenic	Novel	nd	nd
										Maternal	Missense	c.808_812del	p.Gly270Hisfs*9	Pathogenic	Novel	nd	Maxwell et al. (2016) and Pronicka et al. (2016)
12	M	4.93	Yes	Alive	nd	XLP2	*XIAP*	NM_001204401.1	hemi	Maternal	Frameshift	c.898delT	p.Cys300Alafs*8	Pathogenic	Novel	nd	Nd
13	M	4.35	Yes	Alive	Not detected	XLP1	*SH2D1A*	NM_002351.4	hemi	Maternal	Nonsense	c.163C > T	p.Arg55*	Pathogenic	rs111033623	nd	Coffey et al. (1998)
14	M	3.14	Yes	Alive	not detected	XLP2	*XIAP*	NM_001204401.1	hemi	Maternal	Missense	c.655G > A	p.Glu219Lys	VUS PM2 PP3	Novel	nd	nd
15	M	1.25	No	Death	nd	XLP1	*SH2D1A*	NM_002351.4	hemi	Maternal	Splice-site	c.137 + 1_137 + 4del	p.?	VUS PM2 PP3	Novel	nd	nd
16	M	17	No	Alive	nd	XMEN	*MAGT1*	NM_032121.5	hemi	Maternal	Frameshift	c.247delA	p.Arg83Aspfs*3	Pathogenic	Novel	nd	nd
17	F	0	Yes	Alive	Low NK activity	MKD	*MVK*	NM_001114185.3	hmz	Both	Nonsense	c.1162C > T	p.Arg388*	Pathogenic	rs104895360	2.65*e*−05	Prasad et al. (2012)
Patients with monoallelic defects																	
1	F	0.04	No	Death	nd	FHL3	*UNC13D*	NM_199242.2	htz	Not determined	Splice-site	c.753 + 1G > T	p.Ile229Thrfs*35	PATHOGENIC	rs201908137	4.38*e*−5	Alsina et al. (2014)
2	M	0.03	Yes	Alive	Low NK activity, abnormal degranulation	FHL3	*UNC13D*	NM_199242.2	htz	Paternal or maternal	Frameshift	c.2346_2349del	p.Arg782Serfs*12	Pathogenic	rs764196809	1.03*e*−4	zur Stadt et al. (2006)

*hmz* homozygous, *htz* heterozygous, *hemi* hemizygous, *nd* not determined

Our study revealed a predominance of *UNC13D* variations in the Polish population with the majority of frameshift, splice-site and nonsense changes and only one missense variant. Pathogenic variants in *UNC13D* were found in 11 patients with two recurrent variants identified. Three patients were homozygous for a *UNC13D* c.2346_2349delGGAG, p.Arg782Serfs*12 frameshift variant whereas two patients were homozygous for *UNC13D* c.753 + 1G > T splice donor splice-site variant, resulting in exclusion of exon 9. The remaining patients were compound heterozygotes, except for two patients: one female with donor splice-site variant and a male with frameshift variant, who had only one heterozygous change revealed, but young age, severity of symptoms and abnormal degranulation suggested the FHL3. Two novel pathogenic changes: an acceptor splice site c.2710-1G > A and a donor splice site c.569 + 1G > A were found and two known variants were recurrent: a frameshift c.2346_2349delGGAG and a donor splice-site c.753 + 1G > T which occurred, respectively, among 45% (9/20) and 25% (5/20) of *UNC13D* mutated alleles. FHL2 was diagnosed in two patients only and both carried a novel *PRF1* c.808_812delGGCAG, p.Gly270Hisfs*9 deletion in a compound heterozygotes state. In addition to the deletion, they had likely pathogenic missense changes: one carried a known *PRF1* c.938A > T, p.Asp131Val variant, and the other carried a novel *PRF1* c.284G > T, p.Trp95Leu variant. No change in other FHL associated genes were found in the study group.

Four more patients were diagnosed with XLP; two of them had XLP1 caused by different hemizygous changes in *SH2D1A*: a previously described pathogenic nonsense variant: c.163C > T, p.Arg55* and a novel intronic deletion: c.137 + 1_137 + 4delGTGA, classified as VUS according to ACMG with PM2 and PP3 rules. Novel pathogenic variants were also found in *XIAP* in two males diagnosed with XLP2: a pathogenic *XIAP c*.898delT, p.Cys300Alafs*8 frameshift variant and a *XIAP* c.655G > A, p.Glu219Lys missense variant of unknown significance, with PM2 and PP3 rules of ACMG classification.

Two more defects have been identified in genes not related to the familial HLH: a novel predicted pathogenic *MAGT1* c.247delA, p.Arg83Aspfs*3 hemizygous deletion identified in a male diagnosed with XMEN and previously described pathogenic homozygous *MVK* c.1162C > T p.Arg388* nonsense variant in a female.

Genetic analyses of family members demonstrated that all identified changes were inherited from parents. No de novo changes were identified. Detailed data of the study of 19 patients with pathogenic or likely pathogenic variants are included in Table 3.

### Genetic Findings and Clinical Presentation in Patients with Primary HLH

All 13 patients with bi-allelic change presented under one year of age, ten of them in the first three months of life. None of the patients had identified triggers for HLH. CNS disease was diagnosed in 50% of patients with FHL (Table 2).

Clinical characteristics of patients diagnosed with FHL2 included early age at onset and lack of perforin (flow cytometry). Both patients had a severe clinical course. One of patients had CNS involvement and died before stem cell transplantation (SCT) because of progression of the disease.

Functional tests were performed in 15 patients with primary HLH. However, only six patients were tested for sIL2R, 14 patients were tested for NK cytotoxicity, 14 for expression of intracellular perforin and 12 for degranulation – mobilization of CD107a. Genetic testing revealed variants in *UNC13D* in six out of seven patients with abnormal degranulation. Genetic testing performed in two patients with complete lack of perforin expression revealed changes in *PRF1*.

One of the two patients diagnosed with XLP1 died during the course of HLH and EBV infection, while the other is alive after SCT. Both patients diagnosed with XLP2 presented with HLH and EBV infection and are alive after SCT.

A female neonate with mevalonate kinase deficiency (MKD) was born prematurely (threatening eclampsia) by 35-year-old Caucasian mother (gravida 6, para 4) via cesarean section. Parents were non-consanguineous. The pregnancy was normal up to about 20 weeks. By week 27, fetal ascites and anemia with non-immune fetal edema were diagnosed. Two intravenous transfusions of PRC were performed at weeks 28 and 31. Family history revealed death of one child 13 days after birth. That pregnancy was also terminated prematurely due to threatening eclampsia, the child died due to an inflammatory condition and pancytopenia. Two children were born alive and healthy and two spontaneous miscarriages occurred.

The child was born in a severe general condition, the Apgar scores at 1, 3, 5 min and 10 min were 6/6/9/9 points, respectively. The neonate required resuscitation and was admitted to neonatal intensive care unit for ventilation support. The abdomen was markedly distensible, the liver was 6 cm under the right costal margin, the spleen was 6 cm below the left costal margin. During hospitalization, recurring episodes of fever, respiratory and circulatory deterioration with periodic fluid accumulation and pronounced swelling, particularly of the limbs and head occurred. She required intubation and replacement of ventilation in the first week of life and then because of numerous apneas and increasing respiratory acidosis and deterioration of the general condition.

At birth, the complete blood count indicated a platelet count of 145,000/mm^3^, WBC 10,940/mm^3^, and hemoglobin at 127 g/L. The initial coagulation test showed the following: APTT of 44.139 s, INR of 1.47, fibrinogen 0.77 g/L. During the follow-up period, a blood count revealed refractory thrombocytopenia, anemia and neutropenia. She received multiple platelet and RBC transfusions, as well as intravenous infusion of IVIG. Due to recurrent clinical symptoms of respiratory, circulatory and digestive insufficiency, repeated episodes of fever, pancytopenia with extremely low granulocyte and platelet counts, genetic studies to detect HLH, metabolic and auto-inflammatory condition were performed. The neonate underwent a bone marrow puncture because of no response to treatment. Bone marrow analysis revealed normal cellularity, normoblastic maturation of erythroid lineage, feature of dysplasia in megakaryocytes, granulocytes with toxic granules and presence of hemophagocytosis. Tandem metabolic test from capillary blood and urinary screening for metabolic disorders by gas chromatography/mass spectrometry revealed increased mevalonic acid. NGS confirmed pathogenic change in *MVK*. She received Dexamethason and Anacinra followed by allo-SCT.

The patient with XMEN disease was diagnosed with Hodgkin’s lymphoma at the age of 17 years. He presented with persistent EBV viremia, chronic lymphadenopathy and splenomegaly. EBV infection was identified as a trigger in all patients with XLP1, XLP2, and XMEN disease and in 46% of patients with sHLH.

### Genetic Findings and Clinical Presentation in Patients with sHLH

In patients with secondary HLH, no pathogenic variants were identified. There were only benign changes or heterozygous variants classified as VUS, and two patients carried genetic changes “in cis”. One of them was a 5-year-old male with recurrent HLH secondary to EBV infection, who had two variants in one *STXBP2* allele, (NM_001272034.1:c.[828-4C > T;1502G > A], p.(?;Arg501Gln)], both inherited from the father. The degranulation test was normal and thus the patient was qualified as secondary HLH. The patient required SCT and is now is alive with 100% donor chimerism. The second was a 1.5-year-old girl, with two variants identified in one allele of *UNC13D* (NM_199242.2:c.[2542A > G;2983G > C] p.(Ile848Leu;Ala995Pro)), both inherited from the mother. However, she relapsed at 5 months of maintenance treatment and required SCT. She is alive with 100% donor chimerism. Finally, a 10-year-old male with HLH secondary to HHV6 infection carried a heterozygous *PRF1* NM_005041.5:c.272C > T, p.Ala91Val variant, which is known to impair lymphocyte cytotoxicity (Chia et al. 2009). The patient achieved remission on the HLH-2004 protocol and is alive. Detailed data of the study of 35 patients with sHLH are included in Tables 2 and 4.

**Table 4:** Clinical characteristics, treatment and outcome of patients with sHLH (*n* = 35)

	sHLH with genetic variants *n* = 3	sHLH not found to harbor any mutation and not genetically tested *n* = 32 (14 + 18)
Age (year)		
Median	5.6	5.4
Average	5.8	6.7
SD	4.4	4.9
Range	1.5–10	0.5 – 18
Sex (M/F)	2/1	17/15
Trigger	EBV – 2	EBV – 14
	HHV6 – 1	HHV6 – 4
		CMV – 1
		Parvovirus – 1
		RSV – 1
		Mycoplasma pn. – 1
		Chlamydia – 1
		Streptococcus pn. – 1
		Unknown – 8
Functional studies		
Low NK activity	1/2	18/24
Abnormal degranulation	nd	7/20
Decreased perforin	nd	2/21
sIL-2R	nd	Nd
Treatment		
HLH-2004	2	23
Dexamethazone + CSA + IVIg		5
Dexamethazone	1	3
IVIG		1
SCT	2	8
Outcome		
Alive without SCT	1	20
Alive after SCT	2	7
Death before SCT	0	4
Death after SCT	0	1

*RSV* respiratory syncytial virus, *CMV* cytomegalovirus, *EBV* Epstein-Barr virus, *nd* not determined

## Outcome

**Fig. 1:**
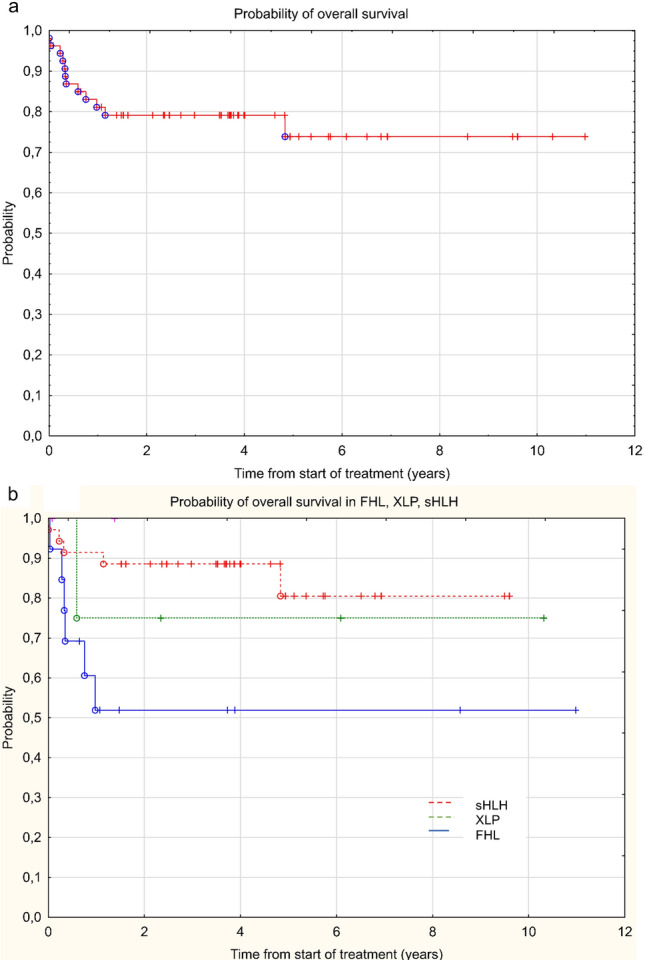
Analysis of survival **a** in the entire group of patients with HLH and **b** in a groups of FHL, XLP and sHLH

Overall survival for the entire group of HLH was 0.74 (SE 0.0934, 95% CI 0.536–0.927) with a median of 3.6 years from follow-up (range 0.03–11 years) (Fig. 1).

The outcome was not significantly different among patients with FHL, XLP and S-HLH and overall survival (OS) was 0.52, 0.76 and 0.81, respectively (*p* = 0.086) (Fig. 2). Twenty-four patients underwent SCT (13 with primary HLH, one patient with MKD and 10 patients with sHLH). The number of patients who achieved complete resolution before SCT were two in the sHLH group and nine in the primary HLH group. All patients achieved engraftment, one died in a short period after SCT. The OS of patients who underwent SCT was 0.71 (SE 0.132, 95% CI 0.454–0.988).

## Discussion

This is the first study investigating the molecular genetics of primary HLH in Poland. Of 54 pediatric patients enrolled in the study over a period of 11 years, 36 received genetic testing. A molecular diagnosis was achieved in 19 (52%) of affected pediatric patients who met HLH criteria and/or displayed abnormal expression of HLH-related proteins or defective cytolytic function.

Thirteen infants had a definite molecular diagnosis of FHL2 or FHL3, based on bi-allelic variants found in all but two, and four patients were diagnosed with XLP1 and XLP2. In addition, one patient with XMEN and one with MKD fulfilled HLH criteria and hence were part of our cohort. The average age of diagnosis of FHL patients was significantly lower (0.16 years) than sHLH patients (6.6 years).

The majority of Polish HLH disease-causing variants were identified in *UNC13D*, and there were: six frameshift, six splicing, three nonsense and one missense variant, among which a frameshift c.2346_2349del and a splice site c.753 + 1G > T were recurrent. All but two of these *UNC13D* variants have been previously reported (Alsina et al. 2014; Murphy 2010; Rudd et al. 2008; Sieni et al. 2011; Yamamoto et al. 2004; zur Stadt et al. 2006), with one of the novel splicing variants (a *UNC13D* c.569 + 1G > A donor splice-site variant) having a single entry in the ClinVar database. Data obtained from different populations have shown ethnic differences in the variation type and frequency of FHL. In European countries, molecular genetics data of FHL are available from a consortium of German, Swedish and Italian studies (Cetica et al. 2010; Rudd et al. 2008; Sieni et al. 2011) where pathogenic variants in both *PRF1* and *UNC13D* have been identified with almost equal frequency (20–30%). Similar proportion of changes were found in the Japanese population, while Korean and Swedish studies revealed a predominance of patients with *UNC13D* changes (Ericson et al. 2001; Ishii et al. 2005; Meeths et al. 2011; Molleran Lee 2004). In turn, studies involving a large cohort of 1892 American patients showed the dominance of changes in *PRF1* (Gadoury-Levesque et al. 2020). In our study, pathogenic variants in *PRF1* were identified in two patients only and both were compound heterozygotes and carried the same deletion of five nucleotides in position 808, which has been reported in two different large cohort studies concerning the molecular background of mitochondrial diseases and breast cancer but have hitherto not been associated with HLH (Maxwell et al. 2016; Pronicka et al. 2016).

Out of eight novel variants identified in the study, six were classified pathogenic or likely pathogenic according to ACMG guidelines (Rehm et al. 2013). One missense variant in the *XIAP* and one splice variant in the *SH2D1A* gene were classified as variants of unknown significance, both with PM2 and PP3 rules. Taking into account the severe course of the disease, the *SH2D1A* c.137 + 1_137 + 4del variant was considered likely to impair the splicing of exon 1 and hence pathogenic. However, cDNA sequencing or SAP expression analysis was not performed due to the inability to collect appropriate samples. The same applies to the other new splicing variants in *UNC13D*, where pathogenicity was not confirmed due to a lack of samples. The possibility of testing patients’ RNA, however, ought to be taken into account, since many pathogenic changes in *UNC13D* affect splicing (Santoro et al. 2008).

Of 13 patients with early-onset HLH, two had only one variant identified. However, both had early onset of HLH and defective lymphocyte exocytosis, which compelled us to qualify them as FHL3 cases. We hypothesize that both patients could be carrying an additional non-coding *UNC13D* aberrations, since pathogenic non-coding aberrations in *UNC13D* frequently cause FHL3. Over 100 different pathogenic or likely pathogenic changes have been identified to date, including deep changes, with the most common *UNC13D* c.118-308C > T (Entesarian et al. 2013; Gadoury-Levesque et al. 2020; Qian et al. 2014; Santoro et al. 2008; Wicki et al. 2016). The deep intronic changes lie in a region, which has not been sequenced routinely and since both patients were sequenced using the Sanger method only, putative deep intronic changes could have been overlooked. Besides deep intronic changes, also a 253-kb inversion in the *UNC13D* has been described as a pathogenic variant causing FHL3 (Qian et al. 2014). But there is also a possibility that patients with mono-allelic pathogenic variants are haploinsufficient for genes involved in perforin-dependent killing function; thus, a mono-allelic defect combined with a powerful trigger is sufficient to cause HLH to develop rapidly (Cetica et al. 2016; Gadoury-Levesque et al. 2020). A congenital form of the disease is usually present in infancy or early childhood (in 80% of cases). However, cases of primary HLH were reported in both fetuses and adults (Clementi et al. 2002; Malloy et al. 2004; Mougiakakos et al. 2012). The adult onset of primary HLH can be caused by mono-allelic changes leading to a partial defect of degranulation or perforin expression. It seems that both pathogenicity of the variant and the strength of a trigger determine the phenotype severity and the time of onset of the disease, hence HLH is consider to have both a dominant and a recessive mode of inheritance (Cetica et al. 2016).

Besides the two FHL3 patients carrying mono-allelic disease-associated *UNC13D* variants that we classified as FHL3, we identified three other patients with monoallelic *STXBP2* and *UNC13D* variants classified as sHLH patients. Two of these required SCT. One of them also carried the *PRF1* p.Ala91Val variant. The change described in 4.6% of the Caucasian population was first considered a polymorphism. However, homozygosity for this common perforin variant is associated with impaired NK cell cytotoxicity in healthy individuals (Vaskoboinik et al. 2007). The homozygous variant is thought to partially impair perforin expression and result in an atypical form, such as late onset of the disease, while the compound heterozygote may result in the typical phenotype of HLH (House et al. 2015). A 10-year-old male with sHLH to HHV6 achieved remission on HLH-2004 protocol and is alive without relapse of the disease. Of the total sHLH cohort, four patients required SCT because of the relapsing course of the disease. There is however a possibility that they had pathogenic changes in HLH-related genes that could have been overlooked during the study, like deep intronic changes or inversion in *UNC13D*. This confirms that a definite distinction between primary and secondary HLH is not possible in many clinical situations. In our case series all patients with malignant and rheumatic diseases were excluded. The most common trigger of sHLH was EBV followed by cytomegalovirus infection.

Since NGS was introduced to diagnosis of HLH, more genetic alterations were found and the spectrum of primary and secondary HLH is constantly changing. According to the Histiocyte Society classification, primary HLH is associated with inherited immune disorders including lymphocyte cytotoxicity defects, pigmentary disorders and abnormalities of inflammasome activation (Emile et al. 2016). This leads to the necessity of sequencing at least *PRF1, UNC13D, STX11, STXBP2, SH2D1A*, *LYST, AP3B1, RAB27A, XIAP* and *NLRC4* genes. HLH-like manifestations may also occur in association with primary immune deficiency and should not be considered as true primary HLH. Primary immunodeficiencies concerning *CORO1A CD27, CTPS1, DOCK8, DOCK2, ITK, MST1, MAGT1, MVK, ORAI1* and *STIM1* gene defects triggered by EBV infection frequently occur in young infants, which results in B lymphoproliferation (Bode et al. 2015). Nevertheless, the list of genes and causative changes identified so far cannot be considered exhaustive. Recently, defects in *RC3H1*, associated with immune dysregulation and systemic hyper-inflammation syndrome, and *RHOG*, associated with defective lymphocyte exocytosis and HLH, have been proposed as additional causes of FHL (Kalinichenko et al. 2021; Tavernier et al. 2019).

Thus, genetic panels need to be updated in line with currents reports.

There is a possibility that a 5-year-old male with recurrent HLH, who had two variants in one *STXBP2* allele (both inherited from the father) could have a form of FHL. However, the degranulation test in his case was normal and the patient was considered to have secondary HLH. There are also examples of dominant negative STXBP2 variants with incomplete penetrance (Spessott et al. 2015).

Auto-inflammatory syndromes are caused by genetic changes in molecules which take part in the regulation of innate immune response. Monogenic inheritance is common. MKD diagnosed in one of our patients is a rare autosomal recessive auto-inflammatory syndrome caused by disease-causing variants in *MVK*, encoding mevalonate kinase. The disease manifests as a continuous spectrum of clinical signs ranging from recurrent febrile episodes commonly accompanied by hepatosplenomegaly, lymphadenopathy, abdominal symptoms, arthralgia and skin rashes, known as hyperimmunoglobulinemia D syndrome, to a more severe form known as mevalonic aciduria, which is also associated with psychomotor retardation, facial dysmorphia, cataract, and failure to thrive. MKD is caused by autosomal recessive variants in *MVK*. In our patient, it was caused by a homozygous *MVK* truncation and the disease presented in intrauterine life with non-immune fetal edema (Georgin-Lavialle et al. 2019).

In conclusion HLH is a heterogeneous syndrome of hyper-inflammation caused by genetic and acquired factors. Historically, patients with HLH are often split into primary and secondary, but with increasing recognition of the clinical diversity of HLH, these division is controversial. The pathogenesis of acquired forms of HLH is not fully understood and it requires further studies. Familial patients are often very young and may not have an obvious infectious trigger. Secondary patients are typically older and usually have a clear environmental trigger. Based on our study we conclude that HLH should be regarded as a clinical culmination of a range of diverse underlying molecular diagnoses as well as environmental challenges, rather than as a distinct disease.

Our study has defined a number of pathogenic variants causative of primary HLH in Polish children.

## Data Availability

Data are available upon request from the corresponding author.
